# Achieving acoustic topological valley-Hall states by modulating the subwavelength honeycomb lattice

**DOI:** 10.1038/s41598-018-35214-9

**Published:** 2018-11-14

**Authors:** Zhiwang Zhang, Ying Cheng, Xiaojun Liu

**Affiliations:** 1 Key Laboratory of Modern Acoustics, Department of Physics and Collaborative Innovation Center of Advanced Microstructures, Nanjing University, Nanjing, 210093 China; 2 State Key Laboratory of Acoustics, Institute of Acoustics, Chinese Academy of Sciences, Beijing, 100190 China

## Abstract

Topological valley-contrasting physics is attracting increasing attention because of its potentials as a promising information carrier in electrics and classical systems. In this work, we reveal the valley-Hall effect and the valley projected edge states in two-dimensional sonic crystals with modulated acoustic honeycomb lattice. The sonic crystals are arranged by soft-material rods and thereby in a sub-wavelength scale, of which the lattice constant is only 0.267 times the wavelength and can be modulated to almost 0.1 times the wavelength. The degenerated valley states are lifted by breaking the inversion symmetry through introducing the refractive-index difference to the rods. The unidirectional excitation of valley chiral bulk state and the non-diffracting Bessel beams are realized by sources carrying orbital angular momentum with proper chirality. Furthermore, we demonstrate that the sub-wavelength valley creation can also be achieved by embedding modulated rubber rods with the mingled steel in a water background, which has significant potential in hydroacoustics, such as underwater communications, sound trapping and directional radiation.

## Introduction

The valley degree of freedom^1–9^, labelling quantum states of energy extrema in momentum space, is attracting growing attention because of its potential as a new type of information carrier. As a result, the concept of valleytronics is proposed^1,2,9^ and many interesting phenomena have been achieved, such as valley selective excitation^10–12^, valley-Hall effects^2,7^ and topologically protected edge states^4,6^. Inspired by this concept, valley-contrasting physics has been theoretically proposed and experimentally observed in photonics^13–19^ and classic airborne acoustic systems^20–31^. The vortex nature of valley states and the topological valley transport in sonic crystals (SCs) were firstly revealed by Lu *et al*.^20–22^, in which the *C*_3v_ symmetry was broken through tuning the orientation of the triangular rod. The topological acoustic delay line based on the reconfigurable topological valley projected edge states (TVPES) has been experimentally observed^23^. The topological valley-Hall phases in a two-dimensional periodic acoustic resonator system was proposed by Yang *et al*.^24^. Recently, the valley-contrasting physics in non-Hermitian systems has been discussed in the artificial acoustic boron nitride^25^. Besides the above valley states in a monolayer SC, the layer-mixed and layer-polarized topological valley-Hall phases in a bilayer SC have also been demonstrated^26^. Here, we propose an approach to achieve valley topological phases in the SC with deep-wavelength scale, in which the lattice constant can be modulated to almost 0.1 times the wavelength. And we also propose the corresponding realization possibility in 2D underwater acoustics, which is essential for various applications.

In this work, we demonstrate that valley-like frequency dispersion can be achieved in a honeycomb SC^32,33^ composed of cylindrical soft-materials rods embedded in air with a sub-wavelength lattice constant, which is only 0.267 times the wavelength. The degenerated valley pseudospin states are lifted by breaking the inversion symmetry through modulating the refractive indices of the unit cells of honeycomb lattice. We also demonstrate that the selected excitation of the valley bulk sates can be obtained by using the circular chiral sources with different polarizations, based on which the triple Bessel beams can be achieved. Topological valley-Hall phase transition and corresponding TVPES are clearly observed. Furthermore, we reveal that identical phenomena can be observed using the rubbers embedded in water, of which the inversion symmetry is broken through modulating rubber rods with the mingled steel. Our work paves the way towards the application possibility of topological acoustic functional devices in topological acoustics, such as acoustic communications and detection.

## Results

### Valley states in the modulated acoustic honeycomb lattice with sub-wavelength lattice constant

**Figure 1:**
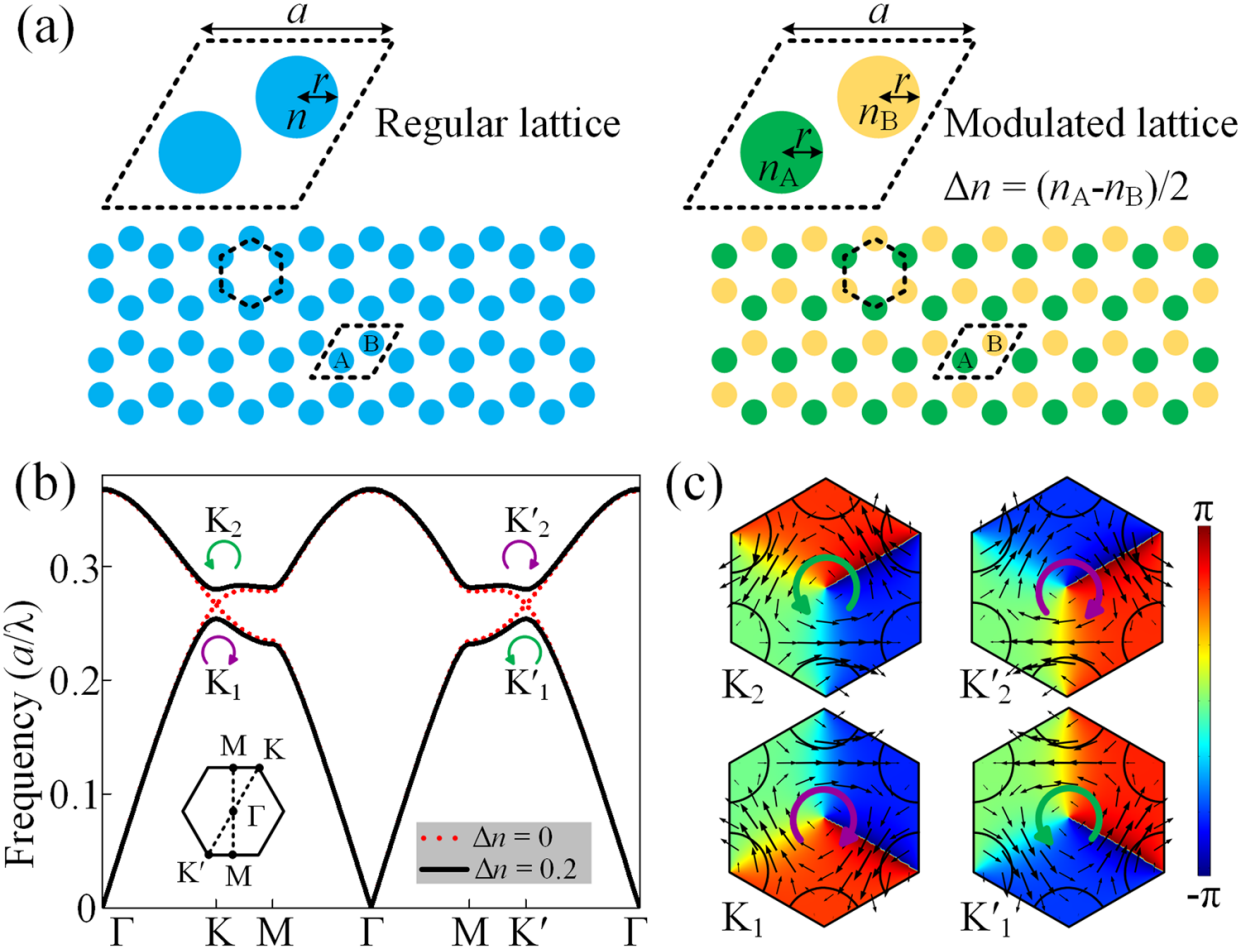
Valley states in the modulated acoustic honeycomb lattice. (**a**) Schematic of the SC (lower panel) and the unit cell composed of two rods embedded in air (upper panel). (**b**) Dispersion relations of the acoustic modes with Δ*n* = 0 and Δ*n* = 0.2. The symbols *K*_1_, *K*_2_, ${K}_{1}^{^{\prime} }$ and ${K}_{2}^{^{\prime} }$ denote the valley states. (**c**) The phase patterns of four valley states. The black arrows show the direction and amplitude of the intensity.

Firstly, we start from the regular acoustic honeycomb lattice [left panel of Fig. 1(a)] composed of isotropic rods embedded in an air background. The lattice constant *a* = √3 cm and the radius of the rod *r* = 0.36 cm. The velocity and density of air are *c*_air_ = 344 *m*/*s* and *ρ*_air_ = 1.21 *kg*/*m*^3^, respectively. The effective refractive index of the rod made of soft materials is defined as $n=\frac{{n}_{{\rm{R}}}}{{n}_{{\rm{air}}}}=3.42$, in which *n*_*R*_ and *n*_air_ represent the refractive indices of the rods and the background air, respectively. Note that we can also employ various different indices by configuring the geometric parameters of the units as shown below. In reality, to achieve this kind of soft acoustic metamaterial, the use of rubber or silica aerogel has been examined theoretically and experimentally^34^. On the other hand, the acoustic pentamode metamaterials can also be a promising candidate, whose effective shear modulus is extremely small compared to its effective bulk modulus^35,36^. It has been proved^32^ that the valley states appear as long as the refractive index satisfies the condition of *n* ≥ 2. Due to the preservation of the inversion symmetry, the single Dirac cone^37^ appears at the corners of the 1st Brillouin zone (BZ) with the frequency of 0.267 *c*/*a*, which is labeled by red dashed curves as shown in Fig. 1(b). Here *c* is the sound velocity in the background. Then, we demonstrate that the perturbation of the system can be achieved through breaking the inversion symmetry by modulating the refractive indices of the rods. As shown in the right panel of Fig. 1(a), the refractive-index difference of two rods in the unit cell, which can be defined as ${\rm{\Delta }}n=({n}_{{\rm{A}}}-{n}_{{\rm{B}}})/2$ with ${n}_{{\rm{A}}}=n+{\rm{\Delta }}n$ and ${n}_{{\rm{B}}}=n-{\rm{\Delta }}n$, is introduced. Owing to the inexistence of the inversion symmetry when introducing ${\rm{\Delta }}n={\rm{0.2}}$, the degenerated valley states are lifted to open a bulk band gap ranging from 0.254 to 0.279 *c*/*a*, as shown in Fig. 1(b). Two pairs of valley sates exist at the *K* and *K*′ points, which possess the intrinsic circular polarized orbital angular momentum. To get an understanding of the physical picture, the distributions of phase and the sound intensity of four valley states are illustrated in Fig. 1(c). The circular propagations of the sound intensity can be viewed as the pseudospins, which are left-handed circular polarized (LCP) and right-handed circular polarized (RCP), respectively. At the *K* point, the acoustic vortex chirality is RCP (LCP) at the higher (lower) state [left panel of Fig. 1(c)]. The counterparts at the *K*′ valley possess invariant vortex but opposite chirality because of the time-reversal symmetry [right panel of Fig. 1(c)].

### Bessel beams achieved by the selective excitation of the inequivalent valley states

**Figure 2:**
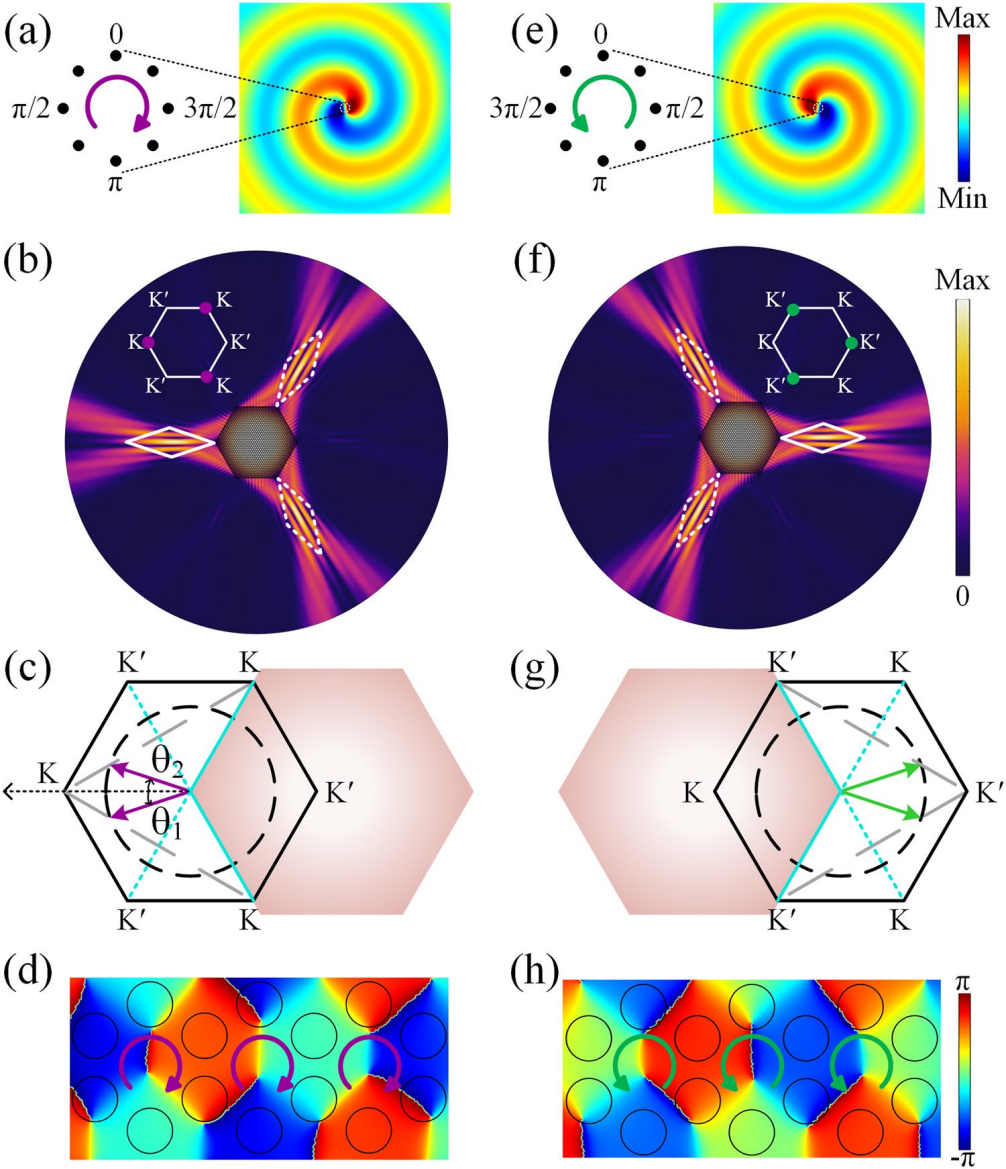
Bessel beams achieved by the selective excitation of the inequivalent valley states. (**a**) Zoomed-in schematic (left panel) and radiated pressure fields in free space (right panel) of the LCP chiral source. (**b**) Distributions of the absolute pressure fields of the triple non-diffracting Bessel beams excited by the central LCP source. White rhombuses represent the BFZ. (**c**) *k*-space analysis on the formation of the left BFZ labeled as the solid white rhombus in (**b**). Black solid hexagon represents the 1st BZ and the black dashed circle shows the dispersion in background. Shadow region represents the SC, of which the cyan lines are for the interfaces. Purple arrows represent the radiation angles out of the SC. (**d**) Phase patterns of the *K*_1_ valley state in the SC when applying the LCP chiral source. (**e**)–(**h**) Corresponding analysis on the formation of BFZ by using the RCP chiral source.

In order to carry information through the valley degree of freedom, the key step is to identify and separate the inequivalent valley states. We demonstrate that the selected excitation of the valley bulk sates can be achieved by external point-like chiral source with proper chirality. For example, the lower valley state at the *K* point with LCP vortex chirality [labeled as *K*_1_ in Fig. 1(c)] can be excited by the point-like chiral source with LCP vortex chirality, as shown in Fig. 2(a). This point-like source consists of a deep sub-wavelength circular array of eight identical point sources with the phase lag *π*/4, of which the phases decrease clockwise. As shown in Fig. 2(b), the LCP chiral source is placed at the center of a hexagon SC with Δ*n* = 0.2, by which the *K*_1_ valley state is excited at the frequency 0.254 *c*/*a* and is out-coupled to the background with *n*_back_ = 1.76. We demonstrate that the direction of the outgoing beam into background depends on the type of excited valley (*K* or *K*′). By matching the parallel component of the incident wavevector on to the equifrequency curve of the background, the propagation direction of the radiated beam can be determined. For simplification, we take the left two sides of the hexagon SC for example, as shown in Fig. 2(c). The theoretical radiation angle can be quantitatively determined by the phase-matching condition ${\boldsymbol{k}}\cdot {e}_{{\rm{side}}}={\bf{K}}\cdot {e}_{{\rm{side}}}$, in which ***k*** and **K** represent the wave vectors in the free space and *K* valley state, respectively; ***e***_side_ is for the base vector along the side. Thus, the radiated directions out-coupled from these two sides can be decided by the two purple arrows as shown in Fig. 2(c), of which the theoretical angle is θ_1,2_ = ±18.23° with the frequency 0.254 *c*/*a*. Two radiative plane beams interference with each other and generate a non-diffracting Bessel beam radiating towards leftmost corner (θ = 0°), of which the explanation is discussed in Supplementary Material Note 1. Similarly, other two Bessel beams can be obtained at the upper-right and lower-right corners. The superposition zone is the Bessel formation zone (BFZ) as labeled by the white rhombus in Fig. 2(b). The phase pattern in the SC shown in Fig. 2(d) further verifies that the *K*_1_ valley state is excited by point-like chiral source with LCP vortex chirality as same as that in Fig. 1(c). The comparison with the BFZ formed by two plane waves is discussed in Supplementary Material Note 2. Note that when the vortex chirality of the source is changed to RCP at the same frequency as shown in Fig. 2(e), the ${K}_{1}^{^{\prime} }$ valley state is excited and three BFZs are formed at the rightmost, upper-left and lower-left corners (Fig. 2(f,g)). Figure 2(h) illustrates the phase pattern in the SC excited by the source with RCP chirality.

### Topological bands inversion

**Figure 3:**
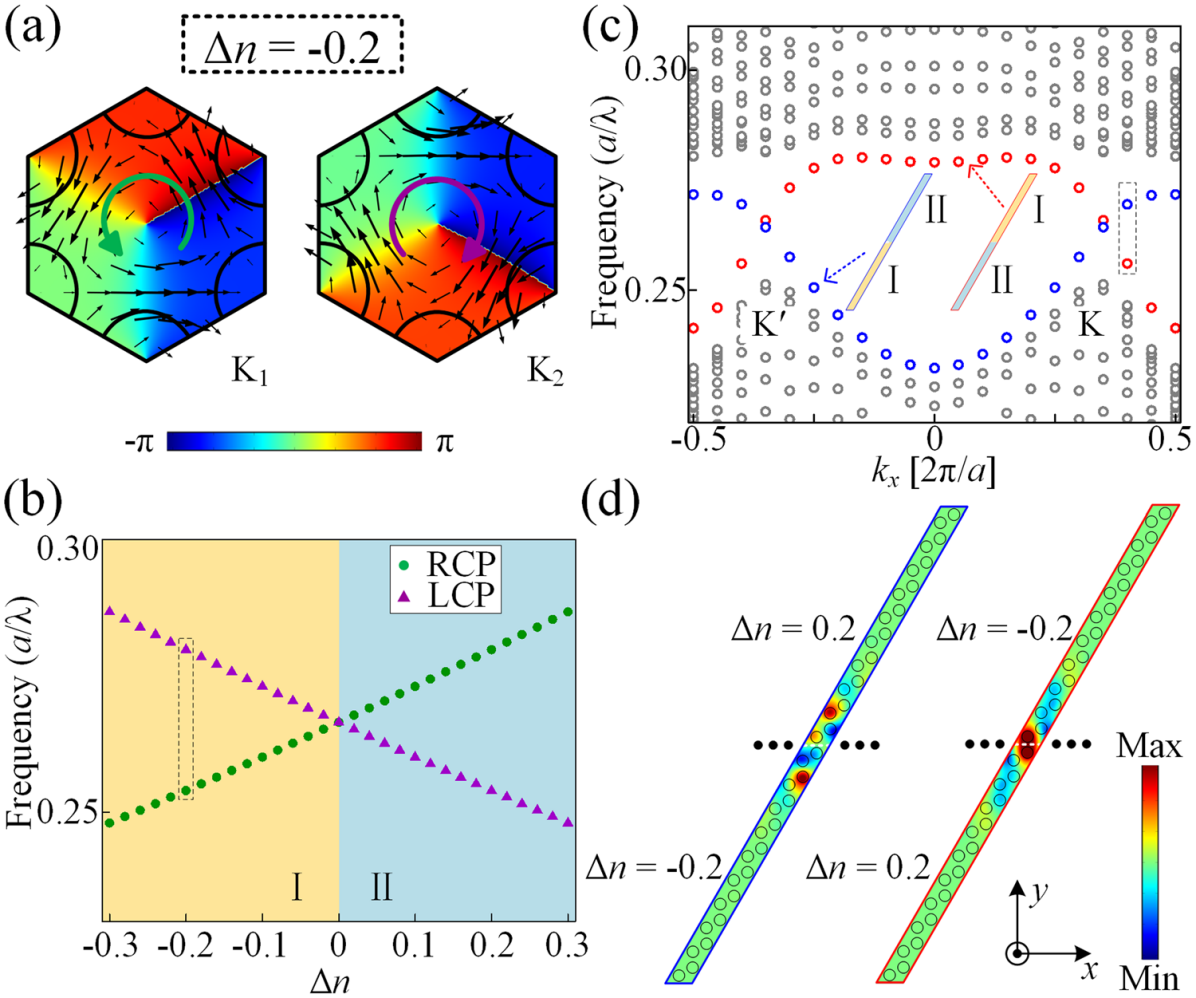
Topological bands inversion. (**a**) Corresponding valley states at the *K* point with refractive-index difference Δ*n* = −0.2. (**b**) Eigenfrequency evolution of the two valley states depending on Δ*n* at the *K* point. The yellow and cyan regions show two different valley-Hall phase, labelled as I and II. (**c**) Dispersion relation of the ribbon-shaped SC with different interfaces (I−II and II−I) comprised of 10 cells on each side. (**d**) Distributions of the total pressure fields at the interfaces shown as the dashed gray frame in (**c**).

The intersection of two different pseudospin valley states takes place in the existence of the inversion symmetry with Δ*n* = 0. We demonstrate that the vortex chirality of each valley state is inverted when the refractive index changes from Δ*n* < 0 to Δ*n* > 0, as shown in Figs 1(c) and 3(a). Figure 3(b) shows that the eigenfrequency of pseudospin valley states separates the band gap at the *K* point as a function of Δ*n*. The green circles and the purple triangles correspond to the RCP and LCP modes, respectively. The band gap is marked with different colors to indicate their different topological characteristics, which are labeled as regions I and II, respectively. The topological invariant is described by the non-vanishing valley Chern indices^6,22,23,38^ $2{{\rm{C}}}^{({\rm{K}},{\rm{K}}^{\prime} )}=\pm \,{\rm{1}}\times {\mathrm{sgn}({\rm{\Delta }}}_{P})$. Here Δ_P_ characterizes the geometrical perturbation induced by Δ*n* and its sign depends on the sign of Δ*n*. As a result, The valley indices of structures I and II are ${{\rm{C}}}_{{\rm{I}}}^{{\rm{K}}}=-\frac{1}{2}$, ${{\rm{C}}}_{{\rm{I}}}^{{\rm{K}}^{\prime} }=\frac{1}{2}$, ${{\rm{C}}}_{{\rm{II}}}^{{\rm{K}}}=\frac{1}{2}$ and ${{\rm{C}}}_{{\rm{II}}}^{{\rm{K}}^{\prime} }=-\frac{1}{2}$. In the setup where the *C*_3_ symmetry is preserved while the inversion symmetry is broken, there is a mass term in the effective Hamiltonian, which can be also considered as the perturbation strength Δ_P_. The topological valley projected edge modes propagate in the presence of domain walls where the mass (Δ_P_) changes sign. Therefore, the interface with different sign of Δ*n* will support the backscattering-immune transmission. As shown in Fig. 3(c), for interface of type I−II labeled by red circles, of which the structure I is on the top and the structure II is on the bottom, there should be a forward-moving edge state at *K*′ point due to ${{\rm{\Delta }}{\rm{C}}}_{{\rm{I}}-{\rm{II}}}^{{\rm{K}}^{\prime} }={{\rm{C}}}_{{\rm{I}}}^{{\rm{K}}^{\prime} }-{{\rm{C}}}_{{\rm{II}}}^{{\rm{K}}^{\prime} }=1$, and a backward-moving edge state at *K* point with ${{\rm{\Delta }}{\rm{C}}}_{{\rm{I}}-{\rm{II}}}^{{\rm{K}}}=-1$. The modes of edge states along the interface of type II−I can be achieved accordingly. Figure 3(d) shows the real-space pressure distributions of the edge states at the typical momenta around the *K* point [labeled as the gray dashed frame in Fig. 3(c)] with *k*_*x*_ = 0.4 × 2π/*a*.

### Topological valley projected edge states

**Figure 4:**
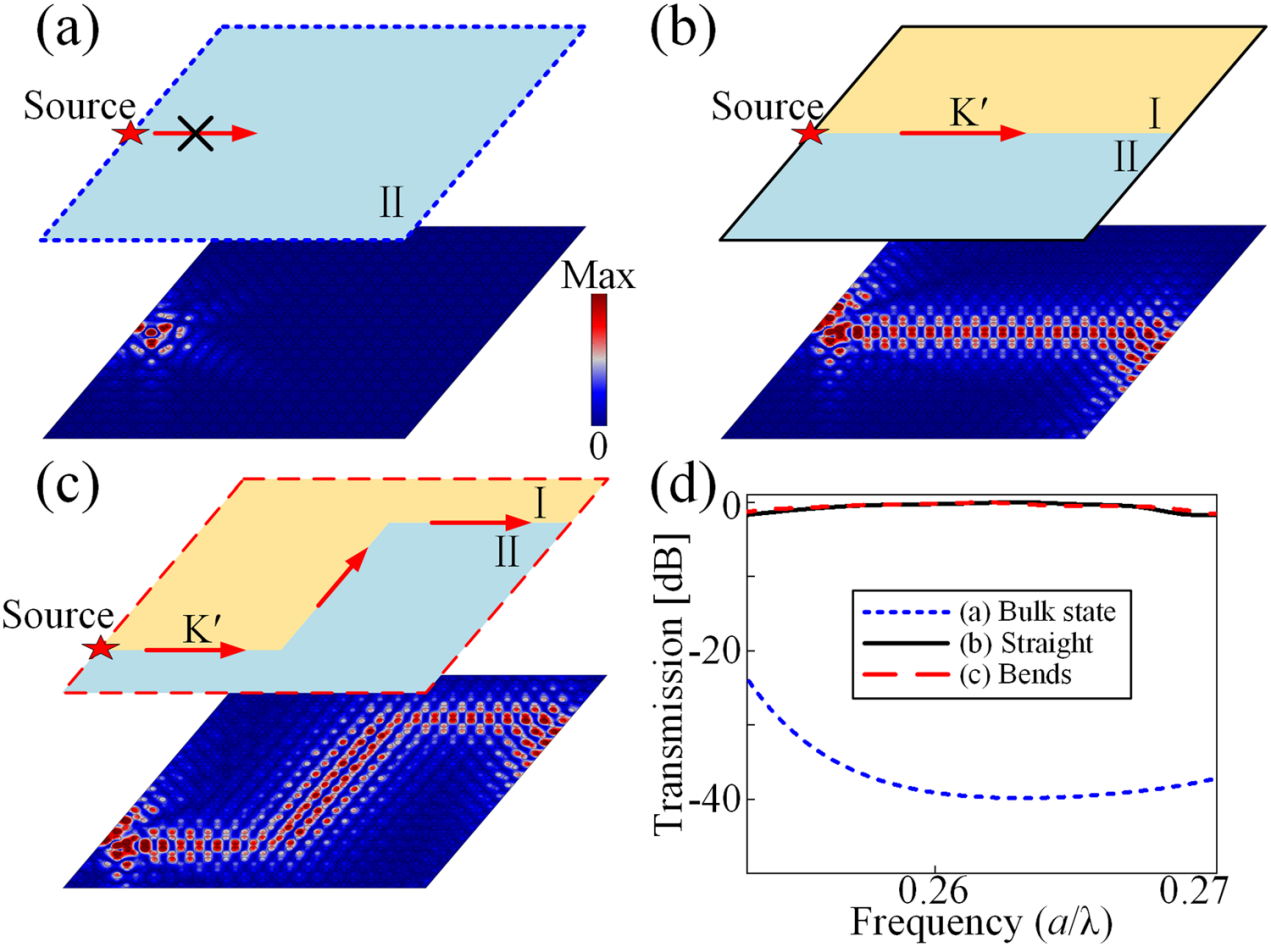
Topological valley projected edge state and its robustness. Distributions of the absolute pressure fields (**a**) through the single-type SC without interface, (**b**) of edge states along the straight interface and (**c**) along the curved path. (**d**) Simulated transmission spectra of topological edge states and bulk state.

We further construct a SC in a finite 20*a* × 20*a* lattice to verify TVPES and its robustness against the curved defects. For the SC composed of the structures with single valley-Hall phase, such as Δ*n* > 0, sound waves excited by the source at the frequency 0.262 *c*/*a*, which is in the band gap, cannot propagate into the SC at all as shown in Fig. 4(a). However, when the straight interface of type I−II is introduced as shown in Fig. 4(b), sound waves projected by *K*′ valley are localized in the vicinity of the interface and decay exponentially away from it, proving that the bulk region is insulating due to the presence of band gaps therein. Figure 4(c) shows a negligible reflection of TVPES with two sharp 60°-bends, which verifies that sharp turns induce very little backscattering. The simulated sound transmission spectra of these three situations within the topological band gap are illustrated in Fig. 4(d), which shows the ~40 dB transmission enhancement of the edge states (black dashed curves) as compared with that of the bulk sate (blue dotted curves), and the robustness of TVPES is verified in the whole band gap. Note that the lattice constant can be tuned into much smaller scale, such as 0.094 times the wavelength, through modulating the refractive indices of the rods (Supplementary Material Note 3).

### Topological creation in a rubber-in-water acoustic system

**Figure 5:**
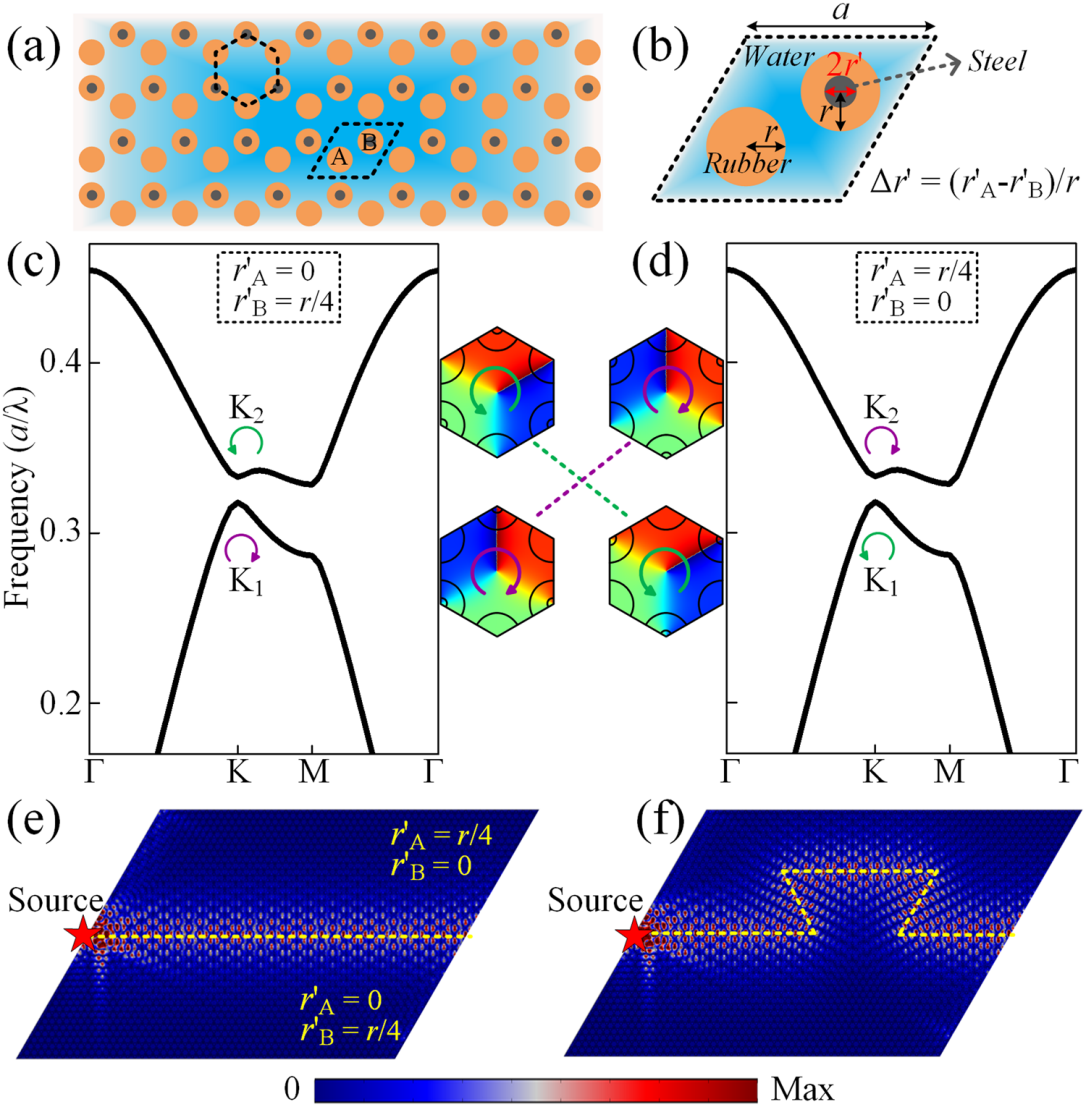
Topological creation in a rubber-in-water acoustic system. (**a**) Schematic of the SC composed of rubber rods embedded in water, which are mingled with steel to tune the refractive indices. (**b**) Enlarged view of the unit cell. Corresponding dispersion relations of the SC with (**c**) *r*′_A_ = 0, *r*′_B_ = *r*/4 and (**d**) *r*′_A_ = *r*/4, *r*′_B_ = 0. Insets: valley-Hall phase inversion underlying the transition between pseudospin states. Distributions of the absolute pressure fields of edge states along the (**e**) straight path and (**f**) curved path.

The above discussions aim to propose a universal theoretical method to achieve TVPES by modulating the refractive indices of the materials, which has been demonstrated in airborne sound. Finally, we also illustrated the corresponding realization possibility of valley-Hall states in underwater acoustics in the same way. Figure 5(a) shows the schematic of the SC composed of rubber rods embedded in water. To break the inversion symmetry, the steel rods is mingled with the rubber to modulate the refractive indices as shown in Fig. 5(b). The mass densities for water, rubber and steel are *ρ*_water_ = 1000 *kg*/*m*^3^, *ρ*_rubber_ = 1300 *kg*/*m*^3^ and *ρ*_steel_ = 7670 *kg*/*m*^3^, respectively. The longitudinal wave velocities in water, rubber and steel are *c*_water_ = 1490 *m*/*s*, *c*_rubber_ = 489.9 *m*/*s* and *c*_steel_ = 6010 *m*/*s*, respectively. The radius of the mingled steel rod is labeled as *r*′. Other parameters are identical with those in Fig. 1(a). Considering the strong mismatch between the longitudinal velocities in these media, the shear wave modes are ignored^39^, which does not affect the topological properties of the system as discussed below. As illustrated in Fig. 5(c), the steel is mingled into atom B but not into atom A with *r*′_B_ = *r*/4, through which the Dirac cone is broken and valley pseudospin states are obtained. Although the dispersion relations are identical when the steel is mingled into atom A but not into atom B with *r*′_A_ = *r*/4 as shown in Fig. 5(d), the inversion of the pseudospin valley states can be observed from the reverted phase patterns of identical valley states. To verify the backscattering-free transmission and the robustness of TVPES against defects in this underwater structure, a point source with at the frequency of 0.322* c*/*a*, which is within the band gap, is placed at the left termination of the straight interface shown in Fig. 5(e) and the curved interface shown in Fig. 5(f). The results demonstrate the existence of the robust topological edge states, which reconfirm the above modulation theory. The valley-Hall phases achieved by introducing radius difference to a rubber-in-water acoustic system are discussed in Supplementary Material Note 4.

## Discussion

In this work, we have demonstrated a universal theoretical method to achieve subwavelength topological valley-contrasting physics. When the inversion symmetry is broken through introducing the refractive-index difference to a regular acoustic honeycomb lattice, the chiral valley states are achieved. We demonstrate the selected excitation of the valley bulk sates and the triple Bessel beams achieved by external point-like chiral source with proper chirality. The Bessel beams have significant potential in developing highly directed and concentrated energy transmission, near-field imaging systems, medical targeting devices and trapping micro-particles^40^. The topological valley phase transition is caused by the non-vanishing valley Chern indices, which ensures the existence of the topological valley-projected edge states. Finally, to verify the generality of the topological creation, we demonstrate that the subwavelength valley-contrasting physics can be also obtained in rubber-in-water system, which have good potential to be used to design underwater delay lines^23,41^ and to radiate directional beam^19,30^. Our results provide the great possibility to the applications of the topological acoustics in communications, medical science and military.

## Methods

### Effective Hamiltonian and the valley-Chern indices

The effective Hamiltonian ${\rm{H}}({k}_{\perp })$, which is a function of the in-plane wavenumber, can be expressed in the basis of the RCP/LCP states in close proximity of the *K* and *K*′ points of the BZ. Derived from the ***k***·P theory, the unperturbed Hamiltonian ${\rm{H}}({k}_{\perp })\equiv {{\rm{H}}}_{0}(\delta {\boldsymbol{k}})$ near the Dirac points can be described as ${{\rm{H}}}_{0}(\delta {\boldsymbol{k}})={v}_{{\rm{D}}}(\delta {k}_{x}{\sigma }_{x}+\delta {k}_{y}{\sigma }_{y})$, where *v*_D_ is the group velocity, *δk* = (*δk*_*x*_, *δk*_*y*_) ≡ *k*_⊥_ − *k*_*D*_ is the distance from the Dirac points, and *σ*_*i*_(*i* = *x*, *y*) are Pauli matrices of the vortex pseudospins. Furthermore, we introduce the perturbation by modulating the refractive indices or the size of rods. The perturbation matrix is diagonalized: ${{\rm{H}}}_{P}={\omega }_{D}{{\rm{\Delta }}}_{P}{\sigma }_{z}$. We can obtain the band structure of the perturbed system by calculating the eigenfrequency Ω(*δk*) ≡ *ω*(*δk*) − *ω*_D_ of the matrix equation ${\rm{H}}(\delta {\boldsymbol{k}})\Psi \equiv {\rm{\Omega }}(\delta k)\Psi$, in which ${\rm{H}}={{\rm{H}}}_{0}+{{\rm{H}}}_{P}$. The sign of perturbation strength Δ_P_ depends on the sign of Δ*n* and Δ*r*. The nontrivial topological properties of the modes can be characterized by the non-vanishing valley-Chern indices. By definition, ${C}^{(v)}={\int }_{\mathrm{BZ}(v)}{d}^{2}\delta k[{\nabla }_{\delta k}\times A(\delta k)]/2{\rm{\pi }}$ with the local Berry connection ${\boldsymbol{A}}(\delta k)=-i{{\rm{\psi }}}_{v}^{\dagger }(\delta {\boldsymbol{k}})\cdot {\nabla }_{k}{{\rm{\psi }}}_{v}(\delta {\boldsymbol{k}})$, where *v* = *K*, *K*′ is the BZ corner. The integral of Berry curvature over the full BZ is zero with Chern number *C* = 0, which is required by time reversal symmetry. However, for small perturbation Δ_P_ the Berry curvature is strongly peaked at the gap minima near *K* and *K*′. As a result, BZ(*v*) is one half of the Brillouin zone. The integral of Berry curvature over an individual valley is accurately defined and the non-vanishing valley-Chern indices can be determined by $2{{\rm{C}}}^{({\rm{K}},{\rm{K}}^{\prime} )}=\pm {\rm{1}}\times {\mathrm{sgn}({\rm{\Delta }}}_{P})$.

### Simulations

Numerical simulations were implemented using COMSOL Multiphysics, a finite-element analysis and solver software. The simulations were performed in the Pressure Acoustic module including the detailed microstructures with actual geometric dimensions. The largest mesh element size was lower than 1/10 of the shortest incident wavelength. Perfectly matched layers were imposed on the exterior of the air domain to eliminate interference from the reflected waves. Cylindrical wave radiation was imposed to achieve the Bessel beams in Fig. 2.

## Electronic supplementary material

Supplementary Material

## Data Availability

The data that support the findings of this study are available from the corresponding author on request.
