# Supplementary material for: Achieving acoustic topological valley-Hall states by modulating the subwavelength honeycomb lattice
Source: Sci Rep. 2018 Nov 14;8:16784. doi: 10.1038/s41598-018-35214-9 (PMC6235864; doi:10.1038/s41598-018-35214-9)
Supplement: Supplementary file 1 — Supplementary Material [file 41598_2018_35214_MOESM1_ESM.pdf]

Achieving acoustic topological valley-Hall states by modulating the subwavelength honeycomb lattice

Zhiwang Zhang,[1] Ying Cheng,[1,2] & Xiaojun Liu[1,2]

[1]*Key Laboratory of Modern Acoustics, Department of Physics and Collaborative Innovation Center of Advanced Microstructures, Nanjing University, Nanjing 210093, China*

[2]*State Key Laboratory of Acoustics, Institute of Acoustics, Chinese Academy of Sciences, Beijing 100190, China*

Correspondence should be addressed to Y.C. (chengying@nju.edu.cn) or to X.L. (liuxiaojun@nju.edu.cn)

Note 1. Formation of the Bessel beams 2

Note 2. The comparison with the BFZ formed by two plane waves 3

Note 3. Tuning the size of the meta atoms into different subwavelength range 4

Note 4. Introducing radius difference to a rubber-in-water acoustic system 5

Note 1. Formation of the Bessel beams

The selected excitation of the valley bulk states can be achieved by external point-like chiral source with proper chirality. As shown in Fig. S1(a), the lower valley state at the K point with LCP vortex chirality [labeled as K_1 in Fig. 1(c)] can be excited by the point-like chiral source with LCP vortex chirality. The valley states refract partly to the free space at the boundary. The theoretical radiation angle can be quantitatively determined by the phase-matching condition $\boldsymbol{k}\cdot\boldsymbol{e}_{\text{side}} = \mathbf{K}\cdot\boldsymbol{e}_{\text{side}}$, in which $\boldsymbol{k}$ and $\mathbf{K}$ represent the wave vectors in the free space and K valley state, respectively; $\boldsymbol{e}_{\text{side}}$ is for the base vector along the side. As a result, the angle θ_1 can be obtained by $|\boldsymbol{k}|\cdot\cos(60°\text{-}\theta_1)=|\mathbf{K}|\cdot\cos 60°$ and $\theta_1 = 60°\text{-arccos}(\frac{c}{3af}) = 18.23°$ with the frequency 0.254 c/a as shown in Fig. S1(a). Figure S1(b) illustrates the simulated result which is coincident with the theoretical prediction. In Fig. S1(c), when the SC boundary is rotated from 60° (seen in Fig. S1(a)) to 120°, which is labeled by the cyan lines, the radiation angle θ_2 can be obtained by $\theta_2 = -18.23°$ as shown in Fig. S1(d). On the other hand, the amplitude distribution of the Bessel beam is proportional to[1]

$$E(\boldsymbol{r}, t) = exp[i(\beta z - \omega t)]\int_0^{2\pi} exp[i\alpha(xcos\varphi + ysin\varphi)]\frac{d\varphi}{2\pi} = exp[i(\beta z - \omega t)]J_0(\alpha\rho), \quad (1)$$

where $\rho^2 = x^2 + y^2$ and J_0 is the zero-order Bessel function of the first kind. From Eq. (1), note that the Bessel beam can be interpreted as a superposition of two plane beams with two opposite traveling angles[1] and it is a nondiffracting beam whose intensity profile decays at a rate inversely proportional to the transverse distance. Thus, as shown in Fig. S1(e), the Bessel beam can be achieved based on the two plane beams with opposite traveling angles as illustrated above. And the superposition zone is the Bessel formation zone (BFZ) labeled by purple color, which is confirmed by the simulated results in Fig. S1(f).

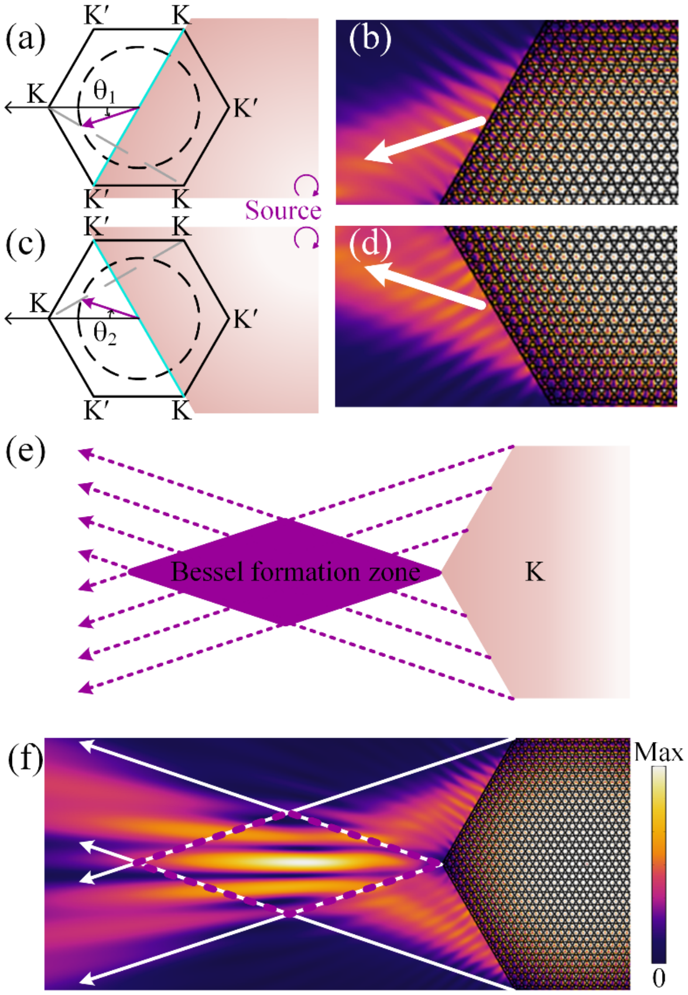

Fig. S1 The *k*-space analysis and the simulated distributions of absolute pressure fields of the radiation directions from the SC with (a)-(b) 60-degree boundary and (c)-(d) 120-degree boundary. Black solid hexagon represents the 1st BZ and the black dashed circle shows the equifrequency contour in background. Shadow region represents the SC, of which the cyan lines are for the interfaces. Purple arrows represent the radiation angles out of the SC. (e) Schematic and (f) simulated distributions of absolute pressure fields of the BFZ.

Note 2. The comparison with the BFZ formed by two plane waves

For comparison, one BFZ is formed by the interface of two plane waves, of which the distributions of absolute pressure fields are shown in Fig. S2(b). The normalized intensity along the longitudinal direction in the middle of the BFZ in both situations is measured as shown in Fig. S2(c). As we can see, although the waveforms are not totally identical, the characteristic

of the Bessel beam can be clearly observed.

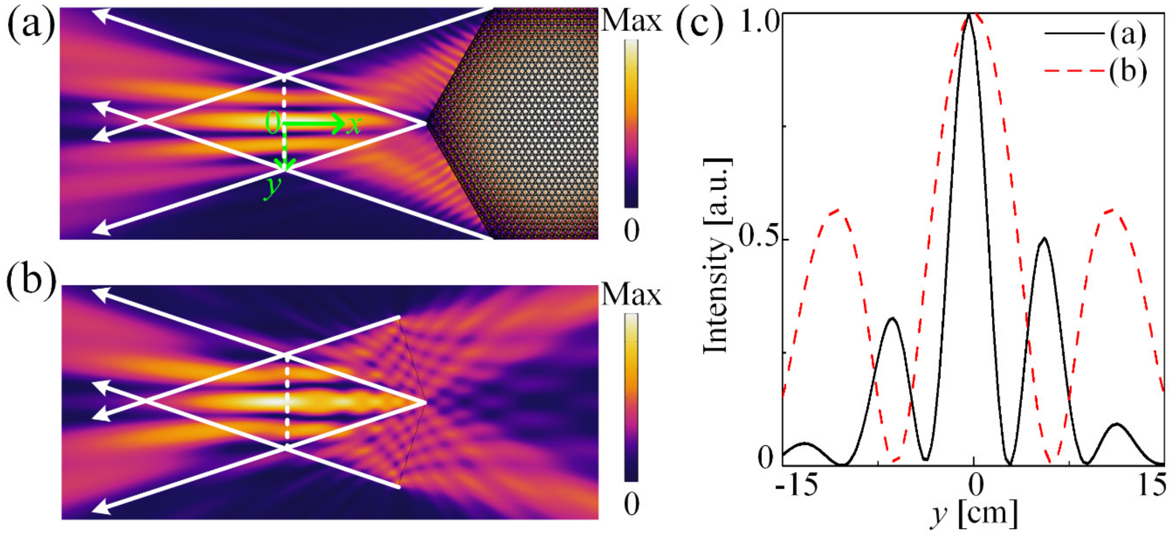

Fig. S2 Simulated distributions of absolute pressure fields of the BFZ (a) formed in the proposed structure and (b) formed by two plane waves. (c) Distributions of the normalized intensity along the longitudinal direction in the middle of the BFZ in both situations, which are labelled by white dashed lines in (a) and (b).

Note 3. Tuning the size of the meta atoms into different subwavelength range

We demonstrate that the size of the meta atoms (the lattice constant of the SC) can be tuned into smaller scale when the refractive indices of the materials increase. As shown in Fig. S3(a), when the refractive indices of the rods increase to $n = 10$, the Dirac cone can be obtained at the frequency of 0.094 c/a. Owning to the inexistence of the inversion symmetry when introducing $\Delta n = 0.2$, the degenerated valley states are lifted to open a bulk band gap. With the different signs of Δn, the valley pseudospins are inverted as shown in Fig. S3(b). Figures S3(c) and S3(d) illustrate the valley projected edge state and its robustness against the sharp bend at the frequency of 0.092 c/a which is in the topological band gap. As a result, the size of the meta atoms can be tuned into 0.094 times the wavelength.

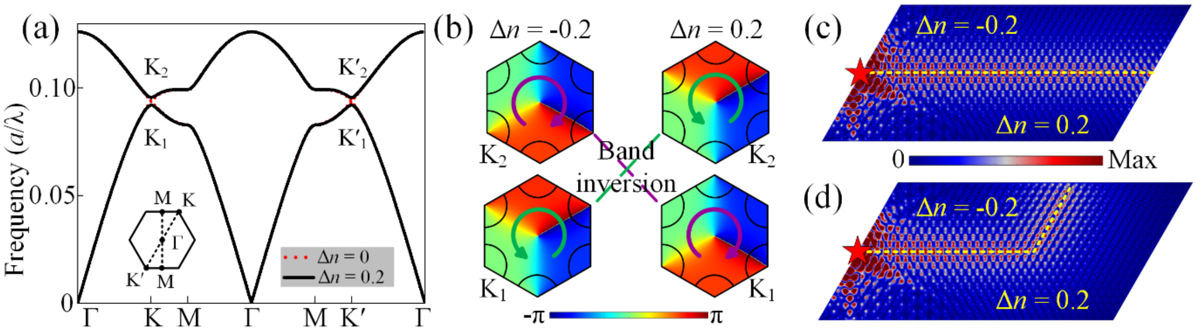

Fig. S3 (a) Dispersion relations of the acoustic modes with $\Delta n = 0$ and $\Delta n = 0.2$, in which the refractive indices of the rods increase to $n = 10$. The symbols K_1, K_2, K_1' and K_2' denote the valley states. (b) Corresponding valley states at the K point with refractive-index difference $\Delta n = -0.2$ and $\Delta n = 0.2$. Distributions of absolute pressure fields of edge states (c) along the straight interface and (d) along the curved path.

Note 4. Introducing radius difference to a rubber-in-water acoustic system

We demonstrate that the topological creation can be also obtained by introducing radius difference to a rubber-in-water acoustic system with unchanged refractive index. Figure S4(a) shows the schematic of the SC composed of rubber rods embed in water, in which the parameters are identical with that in Fig. 5. Instead of changing the refractive indices, the radius difference Δr is introduced here to break the inversion symmetry of the honeycomb lattice as shown in Fig. S4(b). As illustrated in Figs. S4(c)-S4(d), the inversion of the pseudospin valley states in the situation with $\Delta r = 0$ can be observed from the reverted phase patterns of identical valley states. To verify the backscattering-free transmission and the robustness of TVPES against defects in this underwater structure, a point source with the frequency 0.3162 c/a, which is within the band gap, is placed at the left termination of the straight interface shown in Fig. S4(e) and the curved interface shown in Fig. S4(f).

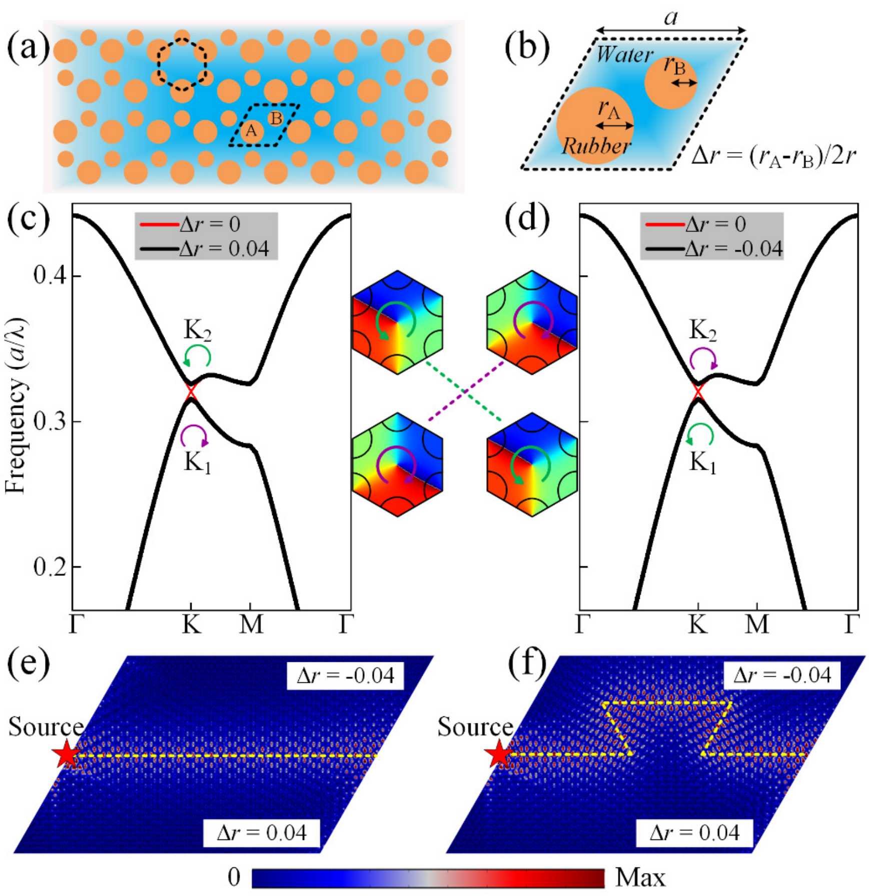

Fig. S4 (a) Schematic of the SC composed of rubber rods embedded in water. (b) Enlarged view of the unit cell. Corresponding dispersion relations of the SC with (c) $\Delta r = 0.04$ and (d) $\Delta r = -0.04$. Insets: valley-Hall phase inversion underlying the transition between pseudospin states. Distributions of the absolute pressure fields of edge states along the (e) straight path and (f) curved path.

References

1. Durnin, J. EXACT-SOLUTIONS FOR NONDIFFRACTING BEAMS .1. THE SCALAR THEORY. *Journal of the Optical Society of America a-Optics Image Science and Vision* **4**, 651-654 (1987).
